# Optimizing Real-Time MI-BCI Performance in Post-Stroke Patients: Impact of Time Window Duration on Classification Accuracy and Responsiveness

**DOI:** 10.3390/s24186125

**Published:** 2024-09-22

**Authors:** Aleksandar Miladinović, Agostino Accardo, Joanna Jarmolowska, Uros Marusic, Miloš Ajčević

**Affiliations:** 1 Institute for Maternal and Child Health-IRCCS “Burlo Garofolo”, 34137 Trieste, Italy; 2 Department of Engineering and Architecture, University of Trieste, 34127 Trieste, Italy; accardo@units.it (A.A.); majcevic@units.it (M.A.); 3 Science and Research Centre Koper, Institute for Kinesiology Research, 6000 Koper, Slovenia; 4 Department of Health Sciences, Alma Mater Europaea University, 2000 Maribor, Slovenia

**Keywords:** motor imagery, BCI, EEG classification

## Abstract

Brain–computer interfaces (BCIs) are promising tools for motor neurorehabilitation. Achieving a balance between classification accuracy and system responsiveness is crucial for real-time applications. This study aimed to assess how the duration of time windows affects performance, specifically classification accuracy and the false positive rate, to optimize the temporal parameters of MI-BCI systems. We investigated the impact of time window duration on classification accuracy and false positive rate, employing Linear Discriminant Analysis (LDA), Multilayer Perceptron (MLP), and Support Vector Machine (SVM) on data acquired from six post-stroke patients and on the external BCI IVa dataset. EEG signals were recorded and processed using the Common Spatial Patterns (CSP) algorithm for feature extraction. Our results indicate that longer time windows generally enhance classification accuracy and reduce false positives across all classifiers, with LDA performing the best. However, to maintain the real-time responsiveness, crucial for practical applications, a balance must be struck. The results suggest an optimal time window of 1–2 s, offering a trade-off between classification performance and excessive delay to guarantee the system responsiveness. These findings underscore the importance of temporal optimization in MI-BCI systems to improve usability in real rehabilitation scenarios.

## 1. Introduction

Brain–computer interfaces (BCIs) are systems that can establish a direct communication pathway between the brain and an external device [1,2]. The potential applications of BCIs span a wide range of fields, including neurorehabilitation, gaming, and communication. In clinical environments, BCIs provide solutions for those with motor impairments [3,4,5,6], enabling the restoration or enhancement of communication [7] and control capabilities [8]

Non-invasive electroencephalography-based BCI is an approach to detect neural potentials from the surface of the scalp. A commonly used system within this area relies on motor imagery (MI), known as motor imagery-based BCI (MI-BCI). MI is the process of visualizing motor actions in the mind without actual physical execution. Within the context of MI-BCI, the technology interprets brainwave patterns linked to this mental practice, involving changes in the brain’s electrical rhythm, known as event-related desynchronization/synchronization (ERD/S) [9], and such brain oscillations, used to classify movements in this study, exhibit a distinct spatiotemporal dynamic [10].

Event-related desynchronization (ERD) refers to a decrease in power within specific frequency bands of the EEG signal, while event-related synchronization (ERS) refers to an increase in power. These changes are typically observed in the alpha (8–13 Hz) and beta (13–30 Hz) frequency bands. During motor imagery tasks, specific areas of the brain exhibit ERD/ERS patterns that correlate with imagined movements. For instance, imagining the movement of the left hand often results in ERD in the right motor cortex and ERS in the left motor cortex, and vice versa for the right hand.

Once the ERD/ERS patterns are decoded, the classifier outputs are used to control external devices or perform specific actions within the BCI system. For example, a BCI user might be able to control a computer cursor, operate a wheelchair, or communicate through a virtual keyboard by imagining different movements.

One of the significant challenges in the widespread acceptance of BCI systems in real-world applications is related to its reliability and complexity in detecting ERD/ERS and other brain patterns [11,12,13,14]. Classification accuracy is the primary measure used to assess the effectiveness of BCIs [2]. Despite advancements, a critical concern in the literature is the lack of emphasis on the responsiveness of BCIs as real-time systems [15]. Studies often prioritize classification accuracy over real-time performance, leading to delays exceeding 4 s in translating EEG signals into actions, impacting user control perception [16]. For real-world applications, especially those requiring immediate interaction like neurorehabilitation or communication aids for individuals with motor disabilities, the delay in processing must be minimized to enhance user experience and functionality [17].

Real-time processing is crucial for BCIs because it directly impacts user experience and system usability. For BCIs to be effective, especially in applications like neurorehabilitation or communication for individuals with motor disabilities, they must translate neural signals into actions swiftly and accurately [17]. Studies on real-time communication systems, such as voice over IP (VoIP), video games, and other machine interactions, indicate that delays exceeding 0.5 s are noticeable and can disrupt user experience [18]. For example, in critical applications like wheelchair control, a delay of 3–4 s would be intolerable as it could lead to significant usability issues and potential safety hazards [19]. Scenarios such as having a control pad for a wheelchair that responds only after 3–4 s or using a keyboard that writes words with such delays highlight the necessity for swift response times in ensuring effective and safe BCI operation [20]. However, it is also true that the MI-EEG spatial patterns change depending on the phase of movement execution or imagery (preparation, execution, or offset). However, longer time windows probably include richer information on these processes, leading to better classification results, and thus, exploration into the trade-off between responsiveness and performance is required [21].

False positives represent another significant issue that reduces the usability of BCIs. A false positive occurs when the system erroneously interprets neural signals as an intention to perform an action. In the context of BCIs, false positives can lead to unintended actions, which can be particularly problematic in critical real-time applications such as wheelchair control. For instance, a BCI controlling a prosthetic limb must avoid unintentional movements that could harm the user or others [20]. High false positive rates can render a BCI unreliable, causing users to lose trust in the system and potentially abandon its use altogether. This perception of the system not working correctly is critical because it undermines the user’s confidence in the technology. In contrast, false negatives, where the system fails to detect an intended action, are less disruptive because the user can repeat the action. Therefore, minimizing false positives is more critical for maintaining user trust and control over the BCI [22]. The false positive rate is particularly significant in critical applications where unintended actions can have severe consequences, such as in the control of medical devices or assistive technologies.

Achieving a delicate balance between high classification accuracy and real-time responsiveness is crucial for the optimal performance of brain–computer interfaces (BCIs) [2,16,23]. Broader time windows in power estimation can indeed enhance accuracy by capturing more data points for feature extraction, as seen in various studies [24,25]. However, this advantage often comes at the cost of reduced responsiveness due to the longer duration needed to collect and process information within these extended windows.

In some BCI studies, the time windows used significantly exceed the acceptable limit, extending up to 4 s [15,26]. However, in real-world applications of BCIs, the excessive delays can lead users to experience a lack of control over the system and therefore significantly diminish the usability and acceptance of BCI technology [27,28,29].

The primary objective of this article is to assess how the duration of time windows impacts performance, measured by classification accuracy and false positive rate, on a real dataset of six post-stroke patients and on the external BCI BCIC IV 2a dataset for study reproducibility analysis. By employing the gold standard Fisher’s Linear Discriminant Analysis (LDA), artificial neural network Multilayer Perceptron (MLP), and linear kernel Support Vector Machine (SVM), our goal is to demonstrate how to optimize and identify the trade-off in the temporal parameters of MI-BCI systems.

## 2. Materials and Methods

### 2.1. Study Population and Protocol

This study involved 6 participants (AS01T–AS06T) who had suffered ischemic strokes (3M/3F 68 ± 8 years), all exhibiting motor deficits. These patients engaged in BCI neurorehabilitation during the early post-acute phase. The inclusion criteria included being a minor/mild unilateral anterior circulation ischemic stroke patient capable of following verbal instructions, effectively communicating, and performing BCI tasks. All patients were recruited from the neurology clinic of Trieste University Hospital in the sub-acute phase (the first two weeks after a stroke). The exclusion criteria were previous brain injuries, the presence of uncontrolled seizures, hemorrhagic stroke, and cognitive impairment measured by Montreal Cognitive Assessment (MoCA) with a score < 24. Moreover, subjects who were not able to participate due to severe aphasia and/or unilateral spatial neglect were also excluded.

Additionally, since this study was conducted on a dataset that cannot be made public due to privacy reasons, to ensure scientific rigor and potential replicability, this study was also performed on nine subjects from the BCI IVa dataset (subjects A01T–A09T). This specific dataset was chosen because it exhibits a similar motor imagery BCI experimental paradigm, enabling possible comparative analysis.

### 2.2. Study Protocol

The BCI performance evaluation utilized data collected during the calibration phase. In this phase, the participants were asked to perform motor imagery while a hand image was displayed on the screen for 4 s, alternating with a blank screen indicating “break.” A fixation cross was presented before the task to prevent eye-movement artifacts caused by the sudden appearance of stimuli on the screen. Each subject had to perform 35–40 trials per session. The type of cueing and imagery provided to subjects is a crucial factor in such studies, as cues have been shown to significantly influence outcomes [30]. However, in this study, to ensure consistency and obtain comparative results, the same cues were used as those applied in the BCI IVa dataset. Moreover, as all patients included in this study were BCI-naive, meaning they had no prior experience with brain–computer interface systems. To ensure proper understanding and execution of the motor imagery tasks, a familiarization phase was conducted prior to the actual EEG recordings. During this phase, patients performed motor imagery exercises without EEG monitoring, using a chronometry technique to guide the timing and execution of the tasks. This familiarization process was intended to help patients become comfortable with the motor imagery tasks and minimize any potential learning curve effects during the EEG-based BCI trials.

### 2.3. EEG Signal Acquisition

**Figure 1:**
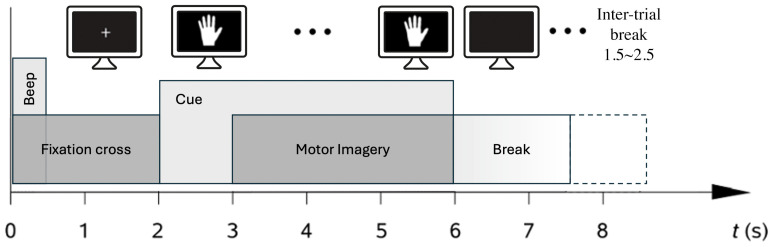
Experimental design of the MI task. After the end of the trial, the experiment looped back to 0 s for the next trial.

MI-elicited EEG data during the calibration of BCI in the post-stroke patients were acquired using a Micromed SAM 32 FO system (Micromed S.p.A., Mogliano Veneto, Italy). Eleven standard Ag/AgCl wet electrodes were used, positioned at FC3, FC4, C4, C3, CP3, CP4, CPz, C2, C4, C6, and C5. The EEG was recorded at a sampling frequency of 256 Hz and filtered from 8 to 30 Hz using a 2nd-order Butterworth bandpass filter. After filtering, the continuous EEG data were segmented from 2 to 6 s relative to the appearance of the fixation cross (see Figure 1). A detailed description of the study protocol and data acquisition for the BCI IVa dataset can be found in [31].

### 2.4. BCI Modeling

The EEG was first filtered (8–30 Hz), and as an additional preprocessing step, the data-driven Common Spatial Patterns (CSP) filter was applied [24]. The extracted features were then input into three classifiers: LDA, MLP, and SVM. These classifiers were selected for their computational efficiency and suitability for real-time applications, as they are known for their fast processing capabilities. LDA was implemented using default parameters without regularization, as it is a widely accepted gold standard in BCI applications for its simplicity and robustness. SVM was configured with a linear kernel and a regularization parameter (C) set to 1.0, which ensures fast convergence and effective separation of the classes without overfitting. MLP, a simple one-hidden-layer model with ten hidden units and the ReLU activation function, was used with a learning rate of 0.01, providing a good balance between quick learning and classification accuracy. The selection of these classifiers and their parameters ensures a practical trade-off between classification performance and real-time usability, making the system well suited for BCI applications where rapid and accurate decision-making is crucial.

Eight distinct, non-overlapping time windows were employed to build the BCI model, capturing various phases of the motor imagery (MI) process. These windows included four shorter windows of 500 milliseconds each, designed to capture rapid neural activity during the initial stages of the MI task, two windows of 1 s to monitor sustained brain activity over a moderate period, one window of 2 s to observe more extended neural patterns, and one window of 4 s to encompass the entire MI process. This combination of time windows allowed for a comprehensive analysis of both short-term fluctuations and sustained patterns in neural activity.

In the post-stroke dataset, classification accuracy was determined by the model’s ability to distinguish motor imagery (MI) tasks from “rest” periods. For the BCI IVa external dataset, classification accuracy was measured by how accurately the model identified left- and right-hand MI tasks. Performance metrics were calculated using two approaches: a 70/30% train/test split and 5-fold cross-validation. During the cross-validation, the dataset was split into five equal parts, with each fold being used as a test set while the remaining four served as the training set. This process was repeated five times, and the mean classification accuracy across all folds was calculated for each combination of time windows, subjects, and classifiers

To calculate classification accuracy, the ratio of correct predictions (both true positives and true negatives) to the total number of predictions was used. True positives referred to the correct identification of MI tasks (or left/right-hand tasks in the BCI IVa dataset), while true negatives referred to the correct identification of “rest” periods (or non-active limb tasks in the BCI IVa dataset). Accuracy thus reflected how well the system performed overall in distinguishing between the different tasks and conditions.

In addition to classification accuracy, the false positive ratio was assessed to evaluate the reliability of the BCI system. A false positive was recorded when the system incorrectly classified a non-task period as an active MI task. For stroke patients, this occurred when the system misclassified a “rest” period as an upper limb motor imagery task, while in the BCI IVa dataset, a false positive occurred when the system incorrectly classified the imagery of the opposite limb. The false positive ratio was calculated by dividing the number of false positive classifications by the total number of predictions made during the “rest” periods for stroke patients or during the imagery of the opposite limb in the BCI IVa dataset.

This metric was crucial in evaluating the system’s propensity to generate unintended actions, thereby affecting the usability and trustworthiness of the BCI system.

## 3. Results

### 3.1. Classification Accuracy

**Figure 2:**
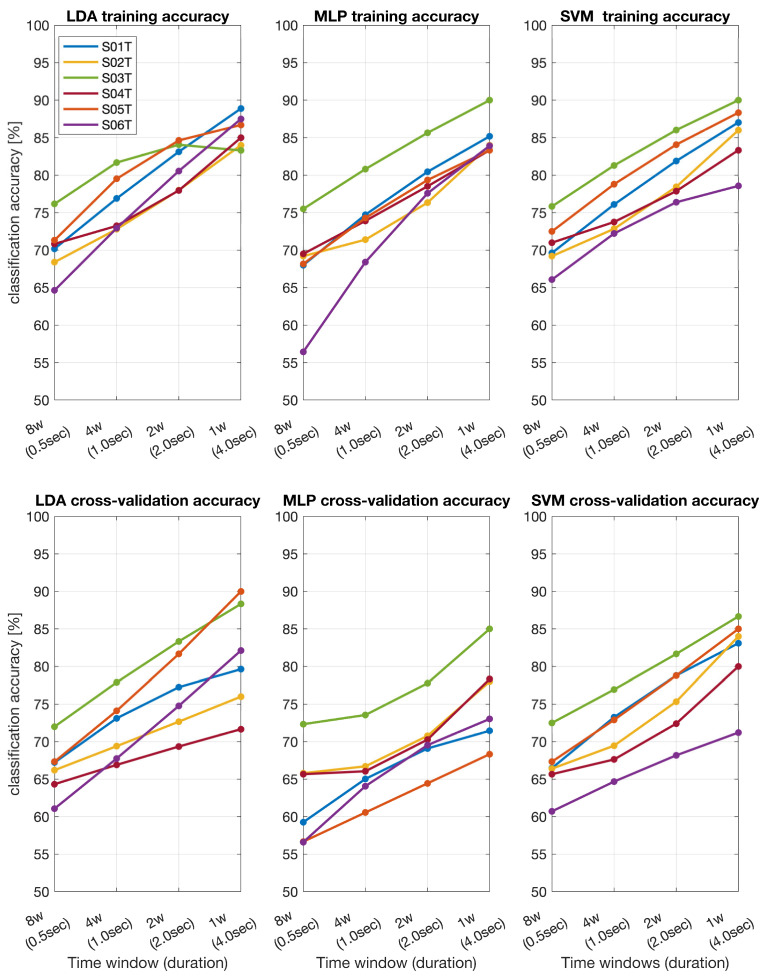
Training and cross-validation classification accuracy across different time windows for stroke patients (tS01T–S06T) obtained by using LDA, MLP, and SVM.

**Figure 3:**
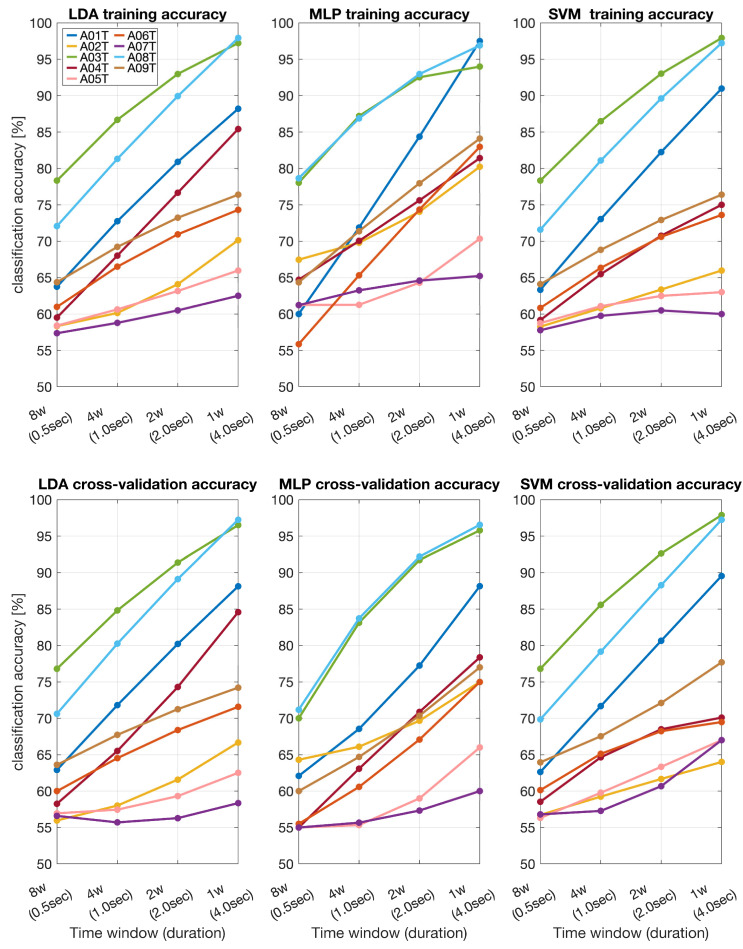
Training and cross-validation classification accuracy across different time windows for the BCI IVa dataset (A01T–A09T) obtained by using LDA, MLP, and SVM.

The classification accuracy for the stroke patients’ dataset (Figure 2) and the BCI IVa dataset (Figure 3) was evaluated using three classifiers: Linear Discriminant Analysis (LDA), Multilayer Perceptron (MLP), and Support Vector Machine (SVM). In the stroke patients’ dataset, all three classifiers demonstrated an increasing trend in accuracy with longer time windows. For the shortest time window (0.5 s), LDA achieved an accuracy range of approximately 65–80%, MLP ranged from 60–85%, and SVM ranged from 70–85%. For the longest time window (4 s), LDA accuracy increased to around 85–100%, MLP to 80–95%, and SVM to 80–95%. Similarly, cross-validation accuracy improved with longer time windows. At 0.5 s, LDA accuracy ranged from 60 to 75%, MLP ranged from 55 to 80%, and SVM ranged from 60 to 75%. At 4 s, LDA accuracy improved to 75–90%, MLP to 70–90%, and SVM to 75–85%.

In the BCI IVa dataset, all three classifiers exhibited a significant increase in accuracy with the length of the time window. At 0.5 s, LDA accuracy ranged from 55 to 85%, MLP from 55 to 80%, and SVM from 55 to 75%. At 4 s, LDA accuracy improved to 80–95%, MLP to 80–95%, and SVM to 75–90%. Cross-validation accuracy also increased with time window length. At 0.5 s, LDA accuracy ranged from 55 to 70%, MLP from 55 to 70%, and SVM from 55 to 65%. At 4 s, LDA accuracy improved to 75–85%, MLP to 70–85%, and SVM to 70–80%.

### 3.2. False Positive Ratio

**Figure 4:**
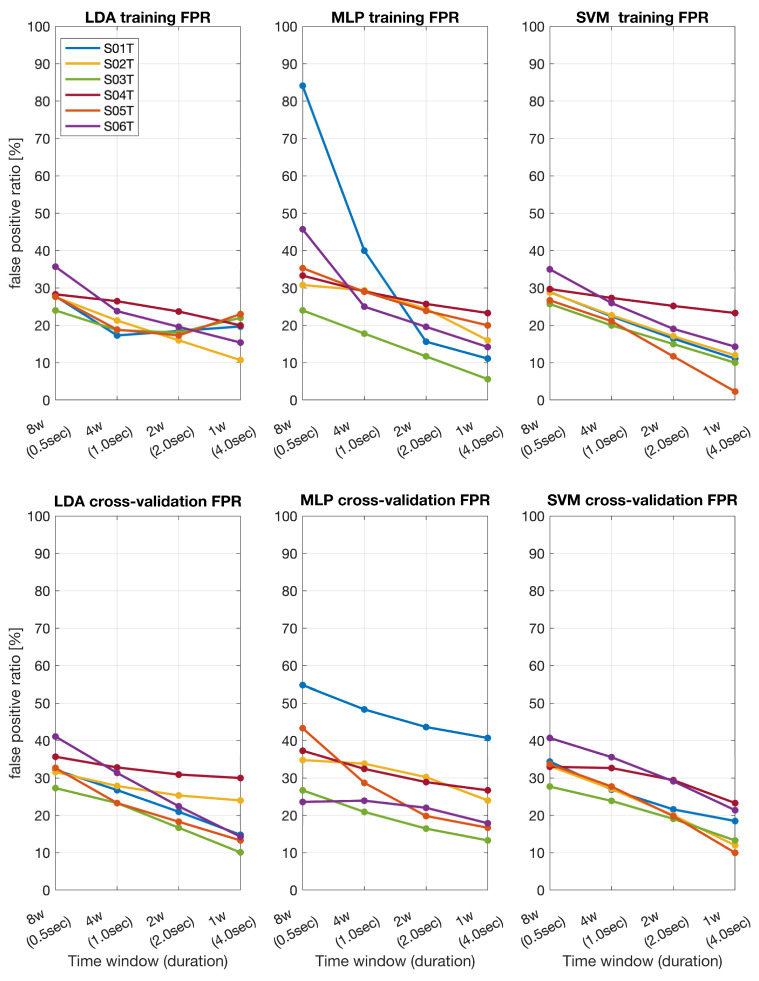
Training and cross-validation false positive ratios (FPR) across different time windows for stroke patients (S01T–S06T) obtained by using LDA, MLP, and SVM.

**Figure 5:**
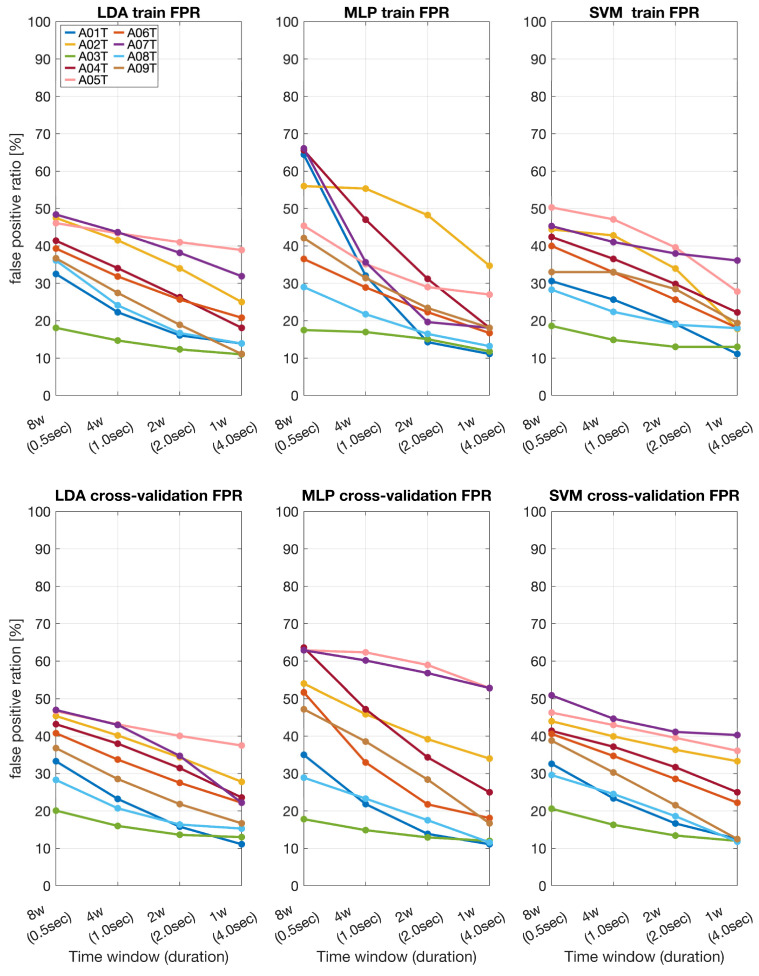
Training and cross-validation false positive ratios (FPR) across different time windows for the BCI IVa dataset (A01T–A09T) obtained by using LDA, MLP, and SVM.

The false positive ratio was calculated to assess the reliability of the BCI system (Figure 4 and Figure 5). In the stroke patients’ dataset, for the shortest time window (0.5 s), LDA showed a false positive ratio of around 20–35%, MLP around 15–35%, and SVM around 20–35%. For the longest time window (4 s), the false positive ratio for LDA decreased to 10–20%, MLP to 10–20%, and SVM to 15–25%. The cross-validation false positive ratios followed a similar pattern. At 0.5 s, LDA exhibited a false positive ratio of 15–25%, MLP 20–40%, and SVM 25–35%. At 4 s, LDA’s false positive ratio reduced to 5–15%, MLP to 15–25%, and SVM to 15–25%.

In the BCI IVa dataset, for the 0.5 s time window, LDA showed a false positive ratio of 20–60%, MLP 15–90%, and SVM 20–50%. For the 4 s window, LDA’s false positive ratio decreased to 10–30%, MLP to 10–30%, and SVM to 10–30%. The cross-validation false positive ratios also decreased with longer time windows. At 0.5 s, LDA had a false positive ratio of 20–45%, MLP 20–50%, and SVM 25–40%. At 4 s, LDA’s false positive ratio dropped to 10–25%, MLP to 10–25%, and SVM to 10–25%.

Overall, both datasets indicated that longer time windows resulted in higher classification accuracy and lower false positive ratios across all classifiers. This trend was consistent for both the training and cross-validation phases. Among the classifiers, LDA generally performed best in terms of balancing accuracy and minimizing false positives, followed by MLP and SVM. These findings underscore the importance of optimizing the time window duration to enhance the performance and reliability of the MI-BCI systems.

## 4. Discussion

Brain–computer interfaces (BCIs) represent an interdisciplinary research frontier, integrating multiple scientific and technological domains [1,2]. By capturing and processing neural signals, BCIs allow direct device control or communication without depending on conventional neural and muscular pathways [2]. These systems have diverse applications, including neurorehabilitation, gaming, and communication, particularly benefiting individuals with motor disabilities. Electroencephalography (EEG), noted for its non-invasive approach, is a common technique in BCIs, capturing electrical patterns from the scalp [32,33].

Despite advancements, the current literature highlights several challenges in adopting BCI technology for real-world applications. Primary concerns include the reliability and complexity of these systems, with significant attention required for their real-time responsiveness [24]. While longer time windows can enhance power estimation accuracy, they often compromise system responsiveness, leading to user dissatisfaction and a perceived lack of control [16,34,35].

For a BCI system to be practical and user-friendly, the maximum acceptable delay in real-time processing should not exceed 1–2 s [19,36]. Delays beyond this threshold can significantly impair the user experience and diminish the perceived control over the interface. Research in human–computer interaction, such as voice over IP (VoIP) and conference calls, supports this finding, indicating that excessive delays are unacceptable as they disrupt the flow of communication and interaction [37].

The primary aim of this study was to assess how the duration of time windows impacts the performance of MI-BCI systems, measured by classification accuracy and false positive ratio, using data from six post-stroke patients and the external BCI BCIC IV 2a dataset [21]. By employing LDA, MLP, and SVM, this study aimed to offer insights on how to optimize and identify the trade-off in the temporal parameters of EEG-based MI-BCI systems and to balance accuracy and responsiveness.

Our results demonstrate that longer time windows generally lead to higher classification accuracy and lower false positive ratios across all classifiers. For the stroke patients’ dataset, LDA achieved the highest accuracy and the lowest false positive ratios, followed by MLP and SVM. This trend was consistent in both the training and cross-validation phases. Similar patterns were observed in the BCI IVa dataset, where longer time windows significantly improved accuracy while reducing false positives.

Considering both accuracy and false positive ratio is crucial for the usability of BCI systems. A high classification accuracy ensures that the system correctly interprets the user’s intentions, while a low false positive ratio minimizes unintended actions, which can be particularly problematic in clinical settings. False positives can lead to erratic device behavior, undermining user trust and the overall effectiveness of the BCI system. Therefore, balancing these two metrics is essential.

A good compromise between accuracy and responsiveness would involve selecting a time window that maximizes accuracy without exceeding the 1–2 s delay threshold for real-time processing. Based on the study’s findings, a time window in the range of 1–2 s appears to offer an optimal balance, providing high accuracy while maintaining acceptable responsiveness. Specifically, a 2 s window is the maximum acceptable delay, but aiming for a 1–2 s window is optimal, as longer delays can disrupt the user experience, similar to findings in human–computer interaction studies [38,39,40]. The movement-related or motor imagery (MI) signals in the EEG, such as brain oscillations used for movement classification in this study, exhibit specific spatiotemporal patterns [10]. The characteristics of these patterns change depending on the type of motor imagery task and vary in length, with spatial patterns shifting depending on the phase of the movement, whether during preparation, execution, or completion, which is also why it is important to prefer shorter time windows. However, longer time windows capture more detailed information about these processes, resulting in improved classification accuracy [10]. Therefore, in the context of rehabilitation, balancing the trade-off between classification speed and accuracy is especially important [21]. In this setting, detection delays can have far more significant consequences than just affecting user experience. Successfully promoting neuroplastic changes depends on precise timing between motor intent and the feedback received. As such, while longer time windows may increase classification accuracy, they may not be as effective in triggering neuroplastic changes as shorter windows.

## 5. Conclusions

In conclusion, our findings indicate that longer time windows generally enhance classification accuracy and reduce false positives across all classifiers, with LDA performing the best. However, to maintain real-time responsiveness, crucial for practical applications, a balance must be struck. The results suggest an optimal time window of 1–2 s, offering a trade-off between classification performance and excessive delay to guarantee the system responsiveness. These findings underscore the importance of temporal optimization in MI-BCI systems to improve usability and reliability in real rehabilitation scenarios.

## Data Availability

Due to patient data privacy concerns, the raw EEG data traces used in this study are not available. However, for the purpose of study replicability, the publicly available BCI Competition IVa dataset has been used and can be accessed for further research and validation.
